# COVID-19 and COVID-19 Vaccinations Lead to Serological Responses in Patients with Inflammatory Bowel Diseases Independent of the Type of Immunomodulatory Medication

**DOI:** 10.3390/biomedicines13092072

**Published:** 2025-08-26

**Authors:** Larissa Kunoff, Martin Kreysing, Annika Gauss

**Affiliations:** Department of Gastroenterology and Hepatology, University Hospital Heidelberg, 69120 Heidelberg, Germany; martin.kreysing@med.uni-heidelberg.de (M.K.); annika.gauss@med.uni-heidelberg.de (A.G.)

**Keywords:** IBD, COVID-19, vaccination, serological response

## Abstract

**Background/Objectives**: The COVID-19 pandemic and the development of vaccines provided the opportunity to monitor disease prevalence and outcomes, vaccinations, their side effects and serological responses in patients with inflammatory bowel disease (IBD). **Methods**: IBD patients of the outpatient clinic at the University Hospital Heidelberg who completed at least one questionnaire on COVID-19 and related vaccinations from July 2021 to August 2022 were included. Spike-IgG antibody titres were determined. Friedman tests, Wilcoxon signed-rank tests and Kruskal–Wallis tests were used for comparisons. The influence of IBD therapy was analysed using linear models with mixed effects. **Results:** The cohort included 520 patients (269 females, mean age = 45.3 years, 60.6% with Crohn’s disease, 35.4% with ulcerative colitis, and 4.0% with unclassified IBD). Four hundred eighty patients (92.3%) received at least one COVID-19 vaccination, and 154 patients (29.6%) were infected by SARS-CoV-2. Among all of them, 94.4% achieved seroconversion. Triple-vaccinated patients with additional SARS-CoV-2 infection developed the highest serological responses (χ^2^ = 16.51, *p* < 0.001, df = 3). An antibody decay over time was observed after the second (*p* < 0.001) and third vaccinations (*p* < 0.001). Regarding individual IBD medications, no differences in mean titres were found after two (χ^2^ = 6.60, *p* = 0.36, df = 6) versus three vaccinations (χ^2^ = 4.97, *p* = 0.42, df = 5). Linear models with mixed effects revealed no influence of IBD therapies on serological responses. **Conclusions**: The highest serological responses were observed in IBD patients after three vaccinations plus SARS-CoV-2 infection without significant differences between IBD therapies.

## 1. Introduction

Early on in the COVID-19 pandemic, researchers started to investigate whether patients with inflammatory bowel diseases (IBDs)—including Crohn’s disease (CD) and ulcerative colitis (UC)—represent a risk group for COVID-19 and whether immunosuppressive therapies for these patients worsen the outcome of SARS-CoV-2 infection [1]. This concern originates from the fact that IBD patients often have compromised immune systems, either as a result of the underlying disease or due to immunomodulatory treatment, potentially predisposing them to the risk of more frequent and more severe infections [1]. Previous studies have reported conflicting findings [1,2,3]. These range from no difference compared to the healthy general population to the influence of different immunosuppressive therapies on the outcome of the disease. Initially, systemic steroids in particular, but also tumour necrosis factor (TNF) inhibitors, stood out as potentially having a negative influence on the outcome of COVID-19 [4,5,6].

With the introduction of vaccines against SARS-CoV-2 infection, the field of research expanded to explore which side effects the vaccinations may trigger in IBD patients and to what extent the serological response to vaccination may be impaired by IBD and related therapies. With regard to the first question, the incidence of side effects to vaccination in IBD patients is comparable to that of the general population. Flares of IBD or severe side effects are very rare [7,8,9]. The answer to the second question cannot definitely be given in the literature. Some studies [10,11,12,13,14] indicate that TNF inhibitors, in particular when used in combination with thiopurines, as well as Janus kinase (JAK) inhibitors, affect the serological response. However, a study by Macaluso et al. (2023) did report an attenuating effect of TNF inhibitors compared to other therapies but concluded that the overall finding of lower antibody titres in IBD patients compared to healthy controls appeared to be largely independent of immunosuppressive treatment [15]. More recent studies continued to observe titre development in relation to the third COVID-19 vaccination. For example, IBD patients showed an increase in serological response after the third vaccination [16,17]. In addition, studies [18,19] show that this increase was weakened by certain immunosuppressive therapies. Here again, TNF inhibitors used in combination with thiopurines and JAK inhibitors are particularly noteworthy. As expected, the titres decrease over time [20]. Infliximab appears to accelerate the decrease in titres [11,21,22]. However, an infection prior to vaccination can also lead to higher serological responses [23].

What has been missing in many of these studies so far is a long-term perspective on COVID-19 infections and vaccinations in IBD patients. The present study addresses this topic by observing IBD patients at Heidelberg University Hospital from July 2021 to August 2022 and recording the prevalence of infections, vaccinations, and side effects of its IBD cohort, and, in a second step, investigating the development of serological responses, both after several vaccinations and in combination with COVID-19 disease (mostly in the context of the Omicron wave after the third vaccination). The study also explored whether IBD therapies had an influence on the development of serological responses.

## 2. Materials and Methods

All study participants were recruited at the IBD outpatient clinic of the University Hospital Heidelberg, which is a tertiary referral centre for the treatment of IBD patients. The study period was from July 2021 to August 2022. Data were retrieved from paper-based standardised questionnaires and electronic records from the clinic information system.

The standardised questionnaires were completed manually by the patients as part of their routine outpatient visits. Information on the history of COVID-19 was requested, including the time point of infection and the need for inpatient or intensive care treatment. Patients were also interviewed on their COVID-19 vaccination course, including the number, dates, and types of vaccinations, as well as side effects and associated IBD relapse symptoms. Completing the questionnaire was expected to take about five minutes on average. Some patients visited the IBD outpatient clinic more than once so that they could complete further questionnaires in case new information on COVID-19 and/or vaccinations was available.

A large part of the data was obtained from the electronic patient records at the clinic information system of Heidelberg University Hospital. Thus various sample characteristics could be extracted in addition to demographic data. At that, it was possible to track which IBD therapies the patients received at the time of vaccinations and infections using the electronic follow-up forms of the IBD outpatient clinic. To minimise errors, the data was checked twice.

Antibody titres against COVID-19 were determined as part of the routine laboratory tests for the IBD patients at the outpatient clinic. Spike-specific IgG antibodies (S1-RBD) were measured using the ADVIA Centaur SARS-CoV-2 IgG (sCOVG) assay (Siemens Healthineers, Munich, Germany), a CLIA targeting IgG to the SARS-CoV-2 spike S1 receptor-binding domain. Results ≥ 1.0 index were considered positive and converted to BAU/mL using the WHO factor of 21.8 BAU/mL per index. Nucleocapsid antibodies were determined using the Elecsys Anti-SARS-CoV-2 assay (Roche Diagnostics, Rotkreuz, Switzerland) to differentiate infection-induced from vaccine-induced seroconversion. All assays were performed according to the manufacturer’s instructions; serum samples were processed promptly or stored at 2–8 °C (≤8 h) or −20 °C (long-term). Titres of patients who visited the outpatient clinic more than once within the indicated time frame were measured repeatedly.

All IBD patients who visited the IBD outpatient clinic for a regular appointment within the indicated time frame were offered to participate in the survey voluntarily. They were free to complete the above-mentioned questionnaire in the waiting room and therein agreed to the evaluation of their data for the purpose of this study.

This prospective, monocentric, uncontrolled observational study has two main objectives: first, to document the occurrence of COVID-19 and vaccinations of IBD patients and note any possible side effects of the vaccinations; second, to describe the development of antibody titres on a descriptive level. The main focus was on S-IgG antibodies, which were measured in BAU/mL and were therefore ratio-scaled variables. The test for S-IgG antibodies turns positive both as a result of infection and of vaccination. Therefore, the available S-IgG antibody titres were analysed inferentially. This included mean comparisons of the antibody titres in relation to the number of vaccinations and COVID-19 infections, as well as in relation to the different IBD medications of the patients. Linear models with mixed effects were applied to analyse potential influences on the S-IgG antibody titres of the vaccinated patients. The JASP (version 0.17.1, University of Amsterdam, Amsterdam, The Netherlands) and RStudio (version 2023.03.0+386, Posit PBC, Boston, MA, USA) programs were used for these analyses, with the former employed primarily for the first part of the analysis plan and the description of the sample.

In the descriptive part of the study, results were indicated either in frequencies and percentages or in mean values, standard deviations, medians and ranges, depending on the type of variable. In the inferential statistical part, various non-parametric test procedures were used depending on the type of mean comparison. The non-parametric methods were chosen because no normal distribution could be assumed for the antibody titres as the target variable. As an additional challenge for this assumption, Kolmogorov–Smirnov tests (RStudio, version 2023.03.0+386) were performed on antibody titres. These differed significantly from the null hypothesis (*p* < 0.05), meaning that a normal distribution could not be assumed. The methods used included the Friedman test (RStudio, version 2023.03.0+386) for comparing several dependent samples, the Wilcoxon signed-rank test (RStudio, version 2023.03.0+386) for comparing two dependent samples, and the Kruskal–Wallis (RStudio, version 2023.03.0+386) test for comparing several independent samples.

In the final step, linear models with mixed effects were used for the calculation in order to better identify the variables influencing the vaccination titres. In the model, a random intercept was assumed for each individual so that respective differences in titre development and discrepancies between the numbers of measurements of individual patients were taken into account. The number of vaccinations was set as a fixed effect, meaning that the analysis factored in whether the measured values were taken under the conditions of a basic immunisation or a booster vaccination. The time interval and the type of immunosuppressive therapy were also assumed to be fixed effects.

## 3. Results

### 3.1. Study Population and Overall Characteristics of the Patients

A total of 537 patients completed the questionnaire at least once. Among these, 14 patients were excluded because the diagnosis of IBD could not be confirmed. Three further patients died during the survey period, so their data were excluded (the cause of death was in no case COVID-19). Thus, 520 IBD patients whose clinical data was recorded as part of their evaluation via the clinic information system of Heidelberg University Hospital had completed at least one questionnaire. Among these 520 patients, *n* = 107 completed the questionnaire on COVID-19 and vaccinations twice, and *n* = 76 at least three times during the survey period. Four hundred and fifty-five patients (85.6%) completed the questionnaires in full. In 420 of the included patients, quantitative determination of S-IgG-AK titres was performed at least once during the study period. Among the 520 included IBD patients, 269 were female, the mean age was 45.3 years (SD = ±15.5), 60.6% suffered from CD, 35.4% from UC, and 4.0% from IBD unclassified (IBD-U). The demographic and clinical characteristics of the study population are shown in Table 1 and Table 2.

### 3.2. Prevalence of Infections, Vaccinations, and Side Effects

Four hundred eighty (92.3%) were vaccinated against COVID-19 at least once, and 154 (29.6%) patients knowingly or unknowingly experienced SARS-CoV-2 infection (23.6% without, 4.9% after the first, 16.0% after the second, 52.1% after the third, and 3.5% after the fourth vaccination). Unknown cases of prior infection were identified based on discrepancies between patients’ self-reported history (denial of prior infection) and the presence of nucleocapsid antibodies. None of the patients reported the need for inpatient treatment. The vaccinated and infected patients showed a seroconversion rate of 94.4% (cut-off: 21.8 BAU/mL). The frequency of side effects and IBD flare symptoms may be viewed in Table 3. The most common side effects mentioned were fatigue (35.2%), discomfort in the vaccination arm (32.4%), headaches (32.4%), and elevated temperature (24.1%). Diarrhoea/increased stool frequency (60.0%), abdominal pain/cramps (42.4%), blood in the stool (15.2%), and worsening of extraintestinal symptoms (15.2%) were mentioned as the most common flare effects. Details on vaccine types, dosing intervals, and homologous versus heterologous regimens are now provided in an additional Table S3.

### 3.3. Serological Responses to Vaccination with or Without Infection

**Figure 1:**
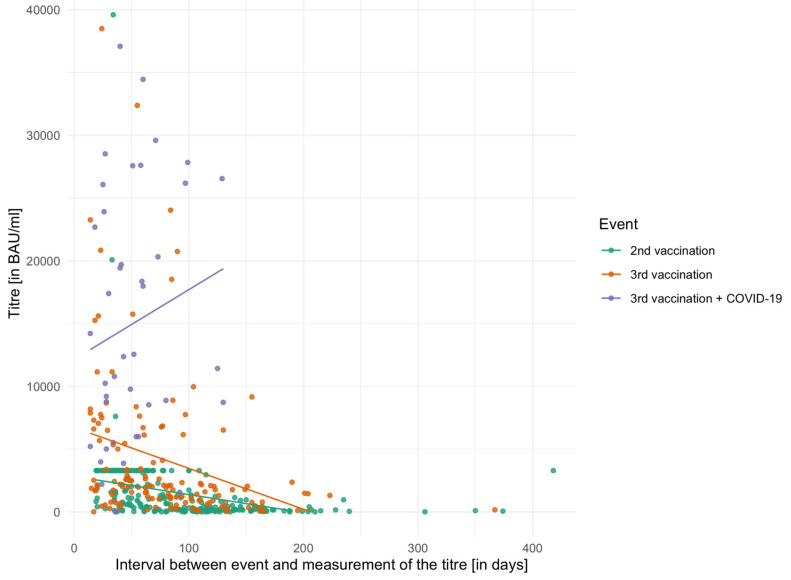
Spike-IgG-antibody titres after every event in relation to the time interval between titre determination and event (vaccination or infection).

The individual mean values of the serological responses of the three event groups (second vaccination, third vaccination and third vaccination with additional COVID-19 disease) are displayed in Table 4. In addition, the last column of the table shows the time interval in days between the last event (vaccination or infection) and the titre determination. In this context, ‘three vaccinations’ denotes a booster dose following a two-dose primary vaccination series. Regarding hybrid immunity, all analyses were performed for infections occurring after three vaccinations, as this subgroup contained the largest number of cases, most of which were recorded during the Omicron wave. Figure 1 shows the vaccination titres of the individual event groups in relation to the time interval between titre measurements. The figure also shows that there was a cap on the titre measurement during the titre survey for the second vaccination and that, therefore, in many patients only a maximum value of 3291.80 BAU/mL could be measured.

The mean titres of the three event groups were examined using the Friedman test and differed significantly from one another (χ^2^ = 16.51, *p* < 0.001, df = 3). In the pairwise comparison of the three groups using the Bonferroni-corrected Wilcoxon signed-rank test, all differed significantly: 2nd vaccination vs. 3rd vaccination (*p* = 0.010), 2nd vaccination vs. 3rd vaccination + COVID-19 (*p* = 0.006), and 3rd vaccination vs. 3rd vaccination plus COVID-19 (*p* = 0.013). The Supplementary Materials include two tables (S1 and S2) presenting serological titres stratified by Crohn’s disease and ulcerative colitis. No statistically significant differences between the two disease groups were detected using the Kruskal–Wallis test, either after the second vaccination (χ^2^ = 0.82, df = 2, *p* = 0.66), the third vaccination (χ^2^ = 0.22, df = 2, *p* = 0.89), or after three vaccinations plus SARS-CoV2 infection (χ^2^ = 0.65, df = 1, *p* = 0.42).

### 3.4. Antibody Titre Decay over Time

For 29 of the included patients, two titre measurements were performed at different time points following the 2nd vaccination. The average interval between these two titre measurements was 78.6 days. In the Wilcoxon signed-rank test, the mean values of the two titres differed significantly from each other (*V* = 370, *p* < 0.001). Comparable results were found for the patients (*n* = 56) in whom two titres were determined after the third vaccination (*V* = 1573, *p* < 0.001). The mean interval between the two measurements was 59.6 days.

### 3.5. Influence of IBD Therapies on Serological Responses

**Figure 2:**
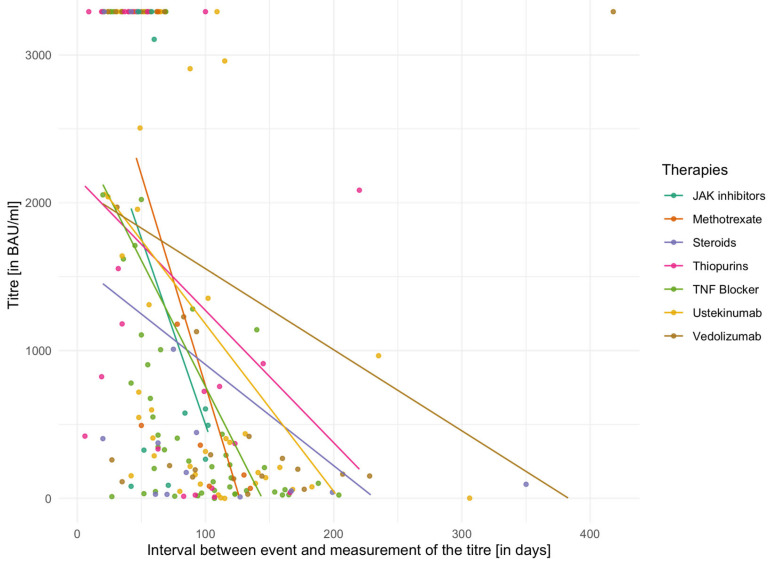
Vaccination titres according to therapy group following the second vaccination. In the figure, the group of Janus kinase inhibitors was adjusted for an outlier (titre at 20,081.70 BAU/mL) in order to represent the scaling more appropriately and to take into account the capping of the remaining vaccination titres. Abbreviations: JAK Janus kinase, TNF tumour necrosis factor.

**Figure 3:**
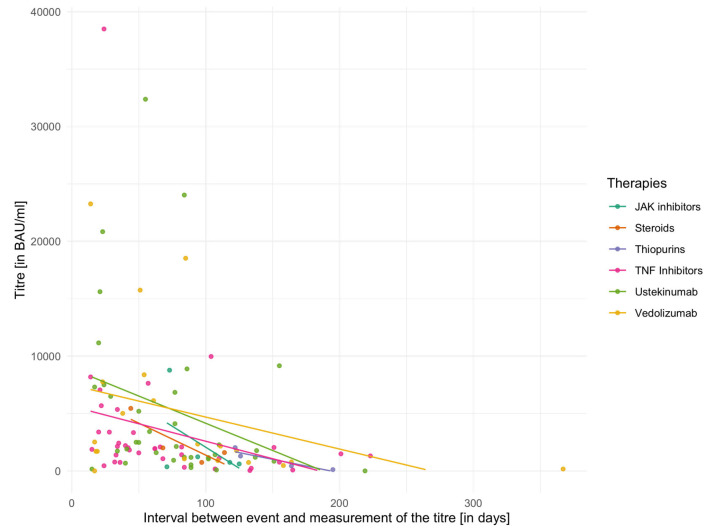
Vaccination titres of the therapy groups after the third vaccination.

The individual mean values of vaccination titres for different IBD medications are shown in Table 5 for the second vaccination and in Table 6 for the third vaccination. In addition, Figure 2 and Figure 3 demonstrate vaccination titres for individual therapies after the second or third vaccination in relation to the interval to titre determination. Here, too, the capping of the titres after the second vaccination has to be considered. The Kruskal–Wallis test did not reveal any significant differences between groups either in relation to the second vaccination (χ^2^ = 6.60, *p* = 0.36, df = 6) or in relation to the third vaccination (χ^2^ = 4.97, *p* = 0.42, df = 5).

A linear model with mixed effects was used to analyse whether the type of immunosuppressive therapy (TNF-inhibitor, ustekinumab, or vedolizumab) or other predictors (time interval between vaccination and titre determination, number of vaccinations) influenced vaccination titres. In this model, the predictors (therapies, time intervals and number of vaccinations) represented the fixed effects, with the assumption of additional random effects for every individual. For the estimation of the model, 197 observations from 157 patients were used. The model showed a significant effect both for the time interval between titre measurement and vaccination (*ß* = −20.42, SE = ±5.59, *p* < 0.001) and for the number of vaccinations (*ß* = 2859.38, SE = ±2626.91, *p* < 0.001). The type of therapy was not significant as a predictor (*ß* = −307.59, SE = ±2290.21, *p* = 0.29). A random intercept was added for individual subjects. Here, the variance was σ^2^ = 5,822,408 (SD = ±2413). Based on the parameters described above, the following model was derived: titre*_i j_* = 5258.949 *−* 318.12 * therapy − 20.65 * time interval + (1∣number of vaccinations*_j_*) +/− (1∣person*_i_*) +ε*_ij_*. It represented the most parsimonious specification, as additional factors such as sex, age, or vaccine type did not demonstrate any significant effects in alternative model configurations.

## 4. Discussion

The aim of this study was to obtain an overview of SARS-CoV-2 infections, vaccinations and antibody titres in the IBD cohort at Heidelberg University Hospital, which represents a mainly difficult-to-treat patient group at a tertiary referral centre. One clear benefit of this study is the establishment of a basis for recommendations regarding vaccinations to be passed on to the patients, most of whom are treated with immunosuppressive medications. In addition, the long-term perspective of the study (data collection period from July 2021 through August 2022) may contribute to the existing research landscape. This large time window made it possible to collect several titres after an event (2nd or 3rd vaccination), allowing sufficient time for titre development. The long observation period also made it possible to record the combination of vaccinations and COVID-19 itself and their influence on antibody titre development in IBD patients.

In the first step of the analyses, it was revealed that 29.6% of the included IBD patients had been infected with SARS-CoV-2 at least once by the end of the survey in August 2022. In comparison, by then, approximately 32.2 million cumulative infections had been registered in the German general population, which represents an approximate share of 38.6% [24]. In addition, a large proportion of patients (92.3%) opted for at least one vaccination and thus stood out from the German general population, with a proportion of 76.4% of people with basic immunisation [25]. It may be interpreted that patients with a serious underlying disease are more health-conscious and therefore more likely to be vaccinated and less likely to enter risky situations with the potential to expose them to severe infection [26]. With regard to possible vaccine side effects, the vaccinated patients in our study reported fewer side effects as compared to the ones described in some other studies [9,27]. A potential explanation may be that our patients had to actively recall their side effects using an open question format, whereas other investigators asked the patients to fill in a predefined list of symptoms. Open-ended questioning is inherently more susceptible to recall bias and underreporting, particularly of mild or transient symptoms, which may not be spontaneously recalled or considered relevant by respondents. The prevalence of IBD flares associated with vaccinations was low and consistent with previous studies [7,8].

In line with results of previous studies [16,17], serological immune responses differed significantly after two and three doses of vaccine. Patients who received three doses of vaccine achieved higher S-IgG antibody titres than those with two doses. Furthermore, patients with additional infection after the third vaccination had an additional significant benefit in terms of antibody development. Similar results have been observed in a healthy population [28]. However, the inclusion of a control group comprising healthy individuals would have enhanced the generalisability of the findings. For IBD patients, Doherty et al. (2023) found a similar effect of previous infection on titre development [23]. Our study supports these data, even though the present study dealt with subsequent infection after the vaccinations, while the other studies dealt with initial infection and subsequent vaccinations.

In the subgroup of our patients with several titres available after every event (second or third vaccination), antibody concentrations decreased significantly over time. This is in line with the findings of various other studies [29,30]. In those studies, however, the examined cohorts consisted of healthy subjects. An implication that arises from this finding is the need for booster vaccinations. Importantly, it must be noted that the immune system does not only consist of IgG antibodies circulating in the blood, but that other components, especially cellular immunity, also contribute to protection against severe SARS-CoV-2 infection after vaccination [31].

Many IBD patients feel uncomfortable about potential detrimental effects of their immunosuppressive therapy on the development of their immune response to vaccination or COVID-19. In response to this need for guidance, several other studies have been published. In particular, TNF inhibitors and their combination with thiopurines and JAK inhibitors have emerged as potential negative influencing factors on titre development [20,21,22]. The present study failed to confirm this observation, as none of the analysed IBD therapies had a particularly detrimental effect on titre development. However, such therapy-specific attenuating effects have been demonstrated in larger multicentre cohorts, including the ICARUS-IBD study, which reported that immunosuppressive treatments can negatively influence serological responses in IBD patients [32]. One possible explanation for this discrepancy is the limited number of patients in certain therapy subgroups, especially JAK inhibitors and thiopurines, which may have reduced the statistical power to detect small effect sizes. Importantly, our analyses regarding therapy subgroups were restricted to patients without prior SARS-CoV-2 infection, and antibody responses were evaluated only after the second or third vaccination. This approach was chosen to avoid confounding by hybrid immunity and to allow for a more homogeneous assessment of vaccine-induced humoral responses. The results of our study provide a further heterogeneous picture regarding the effects of IBD therapies on titre development.

### 4.1. Limitations

In addition to the limitations already discussed in the previous section, the most prominent limitations of this study are of a methodological nature. This is a purely observational study without an experimental approach. As a result, certain parameters were not controllable. For example, the intervals between vaccinations and antibody determination were not standardised, so that only mean values of the time intervals could be specified. One of the major methodological limitations of the study is that changes were made by the laboratory concerning titre measurements after the second vaccination, and titres had to be assumed to reach a certain maximum value of 3291.80 BAU/mL in this study. We continued to be able to detect when titre development was reduced, but not up to what maximum titre development would actually have been possible. This drawback is limited to the period following the second vaccination; the maximum titre increase can also be observed on the basis of the third vaccination or additional SARS-CoV-2 infections, from which realistic values can be inferred. Another limitation is that data on the total clinic census and systematic comparisons between participants and non-participants were not collected, which may limit the assessment of representativeness.

Some of the patients in the different therapy groups may have treated their disease with 5-aminosalicylic acid (5-ASA) preparations. As these are widely used and frequently combined with other therapeutic agents, it was not possible to establish a separate group for this class of medication. Importantly, 5-ASA preparations have immunomodulatory effects, which may also have an influence on titre development. Possibly due to the high proportion of 5-ASA preparations in the treatment of IBD, there are not many studies investigating the individual effect of these preparations on titre development. For example, Casas Deza et al. (2024) found no influence of mesalazine on titre development [33]. However, since 5-ASA preparations were represented in each of the individual therapy groups of the study, we accepted their presence.

There are also limitations concerning the interpretation of data. Our patient cohort is characterised by particularly severe, complex and refractory IBD courses. A more health-conscious behaviour in this special patient group might have resulted in a bias, as it may have influenced infection and vaccination rates as compared to healthy individuals and individuals with less complex disease courses [26]. When interpreting the results, it must also be taken into account that although antibody titres correlate with the responsiveness of the immune system, they say nothing about the risk of infection.

As a final point, it should be mentioned that due to the length of the study period and the rapid further evolution of the virus, several COVID-19 variants emerged during the observation period of the study. Due to the individual characteristics of these variants, the results cannot be automatically extrapolated from one infection to another.

### 4.2. Strengths

By conducting the study at a tertiary treatment centre for IBD, it was possible to observe a large number of patients with moderate-to-severe disease and a high percentage of treatment-refractory courses. It should be acknowledged, however, that a single clinic cannot represent the full spectrum of IBD patients. Above all, this close observation included a long follow-up period during which the titres of individual patients could be recorded several times. Tight monitoring also made it possible to record the combination of vaccinations and COVID-19 itself and to analyse resulting antibody titres. Previously, the combination of triple vaccination and subsequent infection had only been analysed in healthy populations [34]. Beyond that, a large treatment centre such as ours made it possible to observe different immunosuppressive therapies and compare them with one another.

Finally, a central aspect and relevant advantage of this study is that in the context of the SARS-CoV-2 pandemic and the introduction of newly developed vaccines, the opportunity to observe the widespread vaccination coverage of an entire patient group is well utilised. The data illustrates a convenient example of vaccination responses in immunocompromised people, which may also provide lessons to be kept in mind regarding other vaccinations unrelated to COVID-19.

### 4.3. Implications

Several important implications can be drawn from this study. Most notably, vaccination is beneficial. Patients with IBD develop detectable antibody titres and are protected against severe courses of COVID-19. While patients with hybrid immunity exhibit the highest titres, our findings also underscore the importance of booster vaccinations. Nevertheless, serological titres do not fully reflect the overall immune defence, highlighting the need for future research to investigate cellular immunity in IBD patients following vaccination.

## 5. Conclusions

Due to the nature of IBD itself and aggravated by immunosuppressive medications, patients with IBD may be at increased risk of severe SARS-CoV-2 infections and impaired development of serological responses after vaccination against COVID-19. This comprehensive study investigated the prevalence and side effects of COVID-19 and vaccinations as well as spike-IgG-antibody titres in vaccinated and infected patients over the period of a whole year. Our data suggests that IBD patients are not exposed to increased risks (relapse symptoms, side effects, severe courses) in relation to COVID-19 or related vaccinations. The highest serological responses were found in IBD patients with three vaccinations in addition to SARS-CoV-2 infection when compared to double- and triple-vaccinated patients without infection. Further, a significant decay of antibody titres over time was observed. Surprisingly, the study revealed that IBD therapies did not influence antibody concentrations.

## Figures and Tables

**Table 1:** Additional characteristics of the study sample.

	Mean [±SD]	No Information [*n*]
Weight (in kg) Size (in cm) BMI (in kg/m^2^)	74.7 [17.2] 171.5 [12.3] 25.2 [5.0]	5 (1.0%) 19 (3.7%) 19 (3.7%)
Age at diagnosis (in years) IBD duration (in years) Number of previous IBD therapies	29.6 [14.1] 15.8 [11.8] 5.1 [2.4]	2 (0.4%) 2 (0.4%) 7 (1.3%)

Note: Previous therapies include 5-ASA, budesonide, steroids, azathioprine/6-mercaptopurine, methotrexate, tacrolimus, infliximab, adalimumab, golimumab, certolizumab, vedolizumab, ustekinumab, tofacitinib, filgotinib, ozanimod, and ciclosporin. Abbreviations: 5-ASA 5-aminosalicylate, BMI body mass index, IBD inflammatory bowel disease, SD standard deviation.

**Table 2:** Montreal classification of the study sample.

Crohn’s Disease	*n* = 315	Ulcerative Colitis	*n* = 184
A1 A2 A3	42 (13.3%) 216 (68.6%) 56 (17.8%)	A1 A2 A3	22 (12.0%) 118 (64.1%) 44 (23.9%)
L1 L2 L3 L1 + 4 L2 + 4 L3 + 4	86 (27.3%) 45 (14.3%) 149 (47.3%) 5 (1.6%) 4 (1.3%) 23 (7.3%)	E1 E2 E3	4 (2.2%) 55 (29.9%) 118 (64.1%)
B1 B2 B3 B2 + 3 p+ p−	101 (32.1%) 75 (23.8%) 75 (23.8%) 60 (19.1%) 87 (27.6%) 226 (71.8%)		

Note: A = age at diagnosis (A1 = <17 years, A2 = 17–40 years, A3 = >40 years); L = localisation of Crohn’s disease (L1 = ileum, 2 = colon, 3 = ileum and colon, 4 = only upper GI tract); B = type of manifestation (1 = neither stricturing nor penetrating, 2 = stricturing, 3 = penetrating); p = presence of perianal fistulas; E = localisation of ulcerative colitis (1 = rectum, 2 = up to the left flexure, 3 = beyond the left flexure).

**Table 3:** Frequency of reported side effects and flare symptoms after the respective vaccinations.

	1st Vaccination	2nd Vaccination	3rd Vaccination	4th Vaccination
Side effects Flare symptoms	142 (29.8%) 27 (5.2%)	145 (32.4%) 33 (7.4%)	76 (29.0%) 15 (5.7%)	5 (22.7%) 0 (0.0%)

Note: Number of patients who provided information on side effects: *n* = 476 for the 1st vaccination, *n* = 448 for the 2nd vaccination, *n* = 262 for the 3rd vaccination, *n* = 22 for the 4th vaccination; number of patients who provided information on flare symptoms: *n* = 468 for the 1st vaccination, *n* = 48 for the 2nd vaccination, *n* = 263 for the 3rd vaccination, *n* = 22 for the 4th vaccination.

**Table 4:** Average vaccination titres (in BAU/mL) after the respective events (two, three and three + additional infections).

Number of Vaccinations	*n*	Mean	±SD	Median	Range	Days Until Titre Measurement
2 3 3 + COVID	234 140 41	1490.5 (1145.4) 4138.4 (2775.6) 15,028.3 (14,628.3)	3114.1 6047.8 10,162.9	490.1 1991.2 12,370.2	0.00–39,597.7 0.00–38,495.5 0.00–37,083.3	92.4 78.9 51.7

Note: *n* = number of patients, SD = standard deviation; range = minimum to maximum and days until titre measurement indicates the mean of days for every vaccination.

**Table 5:** Vaccination titres (in BAU/mL) of the respective therapy groups after two vaccinations.

Therapy	*n*	Mean	±SD	Median	Range	Days Until Titre Measurement
TNF inhibitor	54	1024.5 (881.8)	1265.6	338.0	0.0–3291.8	84.3
Ustekinumab	47	1257.8 (1165.6)	1324.8	492.1	0.0–3291.8	93.1
Vedolizumab	30	1554.3 (1523.0)	1495.6	773.6	28.3–3291.8	99.9
Thiopurines	22	1470.8 (1430.6)	1370.7	867.4	8.7–3291.8	77.7
Methotrexate	8	1115.4	1389.6	426.5	67.6–3291.8	87.6
Steroids	14	895.5 (769.6)	1325.7	275.1	0.0–3291.8	101.4
JAK inhibitor	11	2928.0 (1338.3)	5843.1	577.0	81.8–20,081.7	68.2

Note: *n* = number of patients; mean: the number in brackets indicates the trimmed mean, i.e., without including the upper and lower 10% of the values; SD = standard deviation; range = minimum to maximum. The group of TNF inhibitors included infliximab and adalimumab, and the group of JAK inhibitors included tofacitinib and filgotinib. Days until titre measurement shows the mean of days for every vaccination. Abbreviations: JAK Janus kinase, TNF tumour necrosis factor.

**Table 6:** Vaccination titres in BAU/mL of the respective therapy groups after three vaccinations.

Therapy	*n*	Mean	±SD	Median	Range	Days Until Titre Measurement
TNF inhibitor	37	3457.1 (2289.5)	6391.1	1941.3	36.40–38,495.5	71.3
Ustekinumab	36	5277.2 (3749.7)	7345.1	2015.8	0.00–32,376.5	76.3
Vedolizumab	20	5055.3 (3697.1)	6661.4	1961.9	0.00–23,261.7	87.0
Thiopurines	4	968.3	863.9	866.9	111.4–2027.8	151.8
Steroids	4	2448.0	2068.3	1801.5	741.2–5448.0	80.8
JAK Inhibitor	5	2340.9	3611.2	740.3	355.6–8776.0	96.2

Note: *n* = number of patients; mean: the number in brackets corresponds to the trimmed mean, i.e., without including the upper and lower 10% of the values; SD = standard deviation; range = minimum to maximum value. The group of TNF inhibitors included infliximab and adalimumab, and the group of JAK inhibitors included tofacitinib and filgotinib. Days until titre measurement shows the mean of days for every vaccination. Abbreviations: JAK Janus kinase, TNF tumour necrosis factor.

## Data Availability

Pseudonymised data is available on reasonable request.

## Supplementary Materials

The following supporting information can be downloaded at: https://www.mdpi.com/article/10.3390/biomedicines13092072/s1, Table S1: Average vaccination titres (in BAU/mL) after the respective events (two, three, three + additional infections) for Crohn’s disease; Table S2: Average vaccination titres (in BAU/mL) after the respective events (two, three, three + additional infections) for ulcerative colitis; Table S3. Vaccination regimens.

## Abbreviations

BMI	body mass index
CD	Crohn’s disease
5-ASA	5-aminosalicyc acid
IBD	inflammatory bowel disease
JAK	Janus kinase
SD	standard deviation
S-IgG antibody	Spike-IgG-antibody
TNF	tumour necrosis factor
UC	ulcerative colitis
