# Supplementary material for: COVID-19 and COVID-19 Vaccinations Lead to Serological Responses in Patients with Inflammatory Bowel Diseases Independent of the Type of Immunomodulatory Medication
Source: Biomedicines. 2025 Aug 26;13(9):2072. doi: 10.3390/biomedicines13092072 (PMC12467913; doi:10.3390/biomedicines13092072)
Supplement: Supplementary file 1 [file biomedicines-13-02072-s001.zip › biomedicines-3789681-supplementary.pdf]

Supplementary Materials

Table S1. Average vaccination titres (in BAU/ml) after the respective events (two, three, three + additional infections) for Crohn's disease

Number of vaccinations	n	Mean	±SD	Median	Range
2	155	1563.2 (1082.7)	3699.2	493.3	0.0 - 39597.7
3	83	4523.1 (2761.5)	7085.8	1969.0	0.0 - 38495.5
3 + COVID	20	16993.7 (16535.8)	11454.8	14976.5	0.0 - 37083.3

Note: n = number of patients, SD = standard deviation; range = minimum to maximum

Table S2. Average vaccination titres (in BAU/ml) after the respective events (two, three, three + additional infections) for ulcerative colitis

Number of vaccinations	n	Mean	±SD	Median	Range
2	67	1342.0 (1273.0)	1379.8	434.0	2.0 - 3291.8
3	54	3477.1 (2749.5)	4133.6	2009.6	0.0 - 20840.6
3 + COVID	21	13156.4 (12936.8)	8624.0	12370.2	0.0 - 28526.6

Note: n = number of patients, SD = standard deviation; range = minimum to maximum

Table S3. Vaccination regimens

1st vaccination	2nd vaccination	3rd vaccination	4th vaccination
**Janssen (n = 17)**			
	+ Comirnaty (n=10)<br>+ Spikevax (n=3)	+ Comirnaty (n=1)<br>+ Spikevax (n=1)	
**Comirnaty (n = 346)**			
	+ Comirnaty (n=346)	+ Comirnaty (n=139)	+ Comirnaty (n=11)<br>+ Spikevax (n=3)
		+ Spikevax (n=55)	+ Comirnaty (n=2)<br>+ Spikevax (n=1)
**Spikevax (n = 31)**			
	+ Spikevax (n = 31)	+ Spikevax (n = 13)<br>+ Comirnaty (n=5)	
**Vaxzevria (n=55)**			
	+ Vaxzevria (n=17)	+ Vaxzevria (n=1)<br>+ Comirnaty (n=9)<br>+ Spikevax (n=1)	+ Comirnaty (n=1)
	+ Comirnaty (n=31)	+ Comirnaty (n=16)	+ Spikevax (n=1)<br>+Novavax (n=1)
		+ Spikevax (n=7)	
	+ Spikevax (n = 7)	+ Comirnaty (n=4)<br>+ Spikevax (n=1)	+ Comirnaty (n=1)

Note. Of the 480 patients who had received at least one COVID-19 vaccination, a complete vaccination schedule could be established for 451. For the remaining patients, either the type of vaccine was not reported or the vaccination schedule was deemed ineligible. The data are presented in a nested manner, such that a patient who received two doses of Comirnaty may also be included in the group of patients who received three doses of Comirnaty.
